# The stifling burden of climate change on African public healthcare systems

**DOI:** 10.3389/fpubh.2025.1559737

**Published:** 2025-05-30

**Authors:** Jack Ogony, Judith Mangeni, George Ayodo, Esther Amulen, Florenc Scopas, Tusubila Juma, Michael Wagaba, Amos Deogratius Mwaka, Lena Nwanja, Sambila Princewill, Ben Oyugi, Arthy Yongo, Sitraka Rakotosolofo, Alberto Francioli, Eunison Mugamu, Julia Davies, Simon Karanja, Corrie Hannah

**Affiliations:** ^1^Department of Environmental Health and Disease Control, Jomo Kenyatta University of Agriculture and Technology, Nairobi, Kenya; ^2^Department of Epidemiology and Biomedical Statistics, School of Public Health, Moi University, Eldoret, Kenya; ^3^Department of Public Health and Community Development, Jaramogi Oginga Odinga University of Science and Technology, Kisumu, Kenya; ^4^School of Public Health, Makerere University, Kampala, Uganda; ^5^Department of Internal Medicine, Faculty of Medicine, Gulu University, Gulu, Uganda; ^6^Department of Environmental Science, University of Buea, Buea, Cameroon; ^7^Department of Economics, University of Antananarivo, Antananarivo, Madagascar; ^8^Department of Geography and Environmental Studies, Stellenbosch University, Stellenbosch, South Africa; ^9^Arizona Institute for Resilience, The University of Arizona, Tucson, AZ, United States

**Keywords:** adaptation, climate change, disaster risk, healthcare system, vulnerability, preparedness, vector-borne diseases, resilience

## Abstract

**Background:**

Climate change is the greatest health threat of the 21st century to global health and primary health care. Despite being the least contributor to global greenhouse gas emissions, Africa is disproportionately facing severe impacts of climate change, particularly on its health systems which is already neglected and underfunded. The crisis poses a fundamental threat to human health by undermining healthcare infrastructure, straining workforce capacity, and diminishing global progress toward universal health coverage. It disrupts the physical environment, natural and human systems, and the functionality of healthcare systems, acting as a multiplier threat that jeopardizes and potentially reverses decades of health gains. The Sendai Framework, a roadmap for making vulnerable and marginalized communities safer and more resilient emphasizes the importance of investing in disaster risk prevention and reduction through both structural and non-structural measures, which are vital for enhancing socio-economic, health, and cultural resilience. This narrative review is based on the insights drawn from Climate Adaptation Research Program scholars across Africa. It explores the current and projected burden of climate change on the continent’s healthcare systems. It underscores the urgent need to integrate climate resilience into healthcare planning, fostering cross-sectoral collaboration, and ensures the sustainability of health systems amid escalating climate challenges.

**Conclusion:**

The impacts of climate change on health represent a significant global challenge, demanding the establishment of robust and resilient healthcare systems. To mitigate the catastrophic and lasting effects of the climate crisis on healthcare and to prevent millions of climate-related deaths, it is essential to enhance resilience and preparedness.

## Background

The severe damages and impacts caused by extreme events in a changing climate are not only hindering progress toward achieving the 2030 Sustainable Development Goals (SDGs) but are also reversing hard-won public health development gains from the past (1). Climate change is the most significant threat to global health today. It is no longer a looming threat but rather a destructive reality with dire predictions for the future. According to the World Health Organization (WHO), there will be an increase of 250,000 more annual deaths between 2030 and 2050 due to the impacts of climate change (2). Africa is particularly vulnerable to climate change (3), with current projections indicating a significant increase in mean temperatures (3°C to 6°C) by the end of the 21st century, exceeding the global average, relative to the reference period of 1986–2005 (4).

By mid-century, mean temperatures across most of Africa are projected to exceed 2°C, potentially reaching 4°C by the end of the century (5). These changes pose significant threats to global health, with the greatest impacts likely to be felt in low-income Countries and communities (6). A health system consists of; all organizations, people, and actions whose primary intent is to promote, restore, or maintain health care services of populations (3), whereas public health aims to prevent illness and injury in entire populations, healthcare facility is to treats individual who are already sick. Although primary healthcare plays a critical role in safeguarding the well-being of the population in the face of these rising health risks, it is currently one of the most critically affected by the extreme climate events (7). Healthcare systems in Africa already suffer from neglect and underfunding, leading to severe setback across the 6 WHO healthcare delivery pillars (8). Instead, the dilapidated healthcare systems in Africa have facilitated medical tourism, forcing patients to leave the continent to seek various forms of attention abroad.

Climate change is not only an environmental crisis but also a health crisis, demanding global actions, effective partnerships and innovative solutions as its impacts become increasingly evident (9). In sub Saharan Africa, continuous climate variability exposes vulnerable and marginalized populations including the older adults, pregnant women, migrants, and children to heightened risks (10). The Sendai Framework for Disaster Risk Reduction 2015–2030 (11) which is a roadmap for making vulnerable and marginalized communities safer and more resilient. The overall objective of the framework is to substantially reduce disaster risks (DRR) and losses in lives, livelihoods, health, economy, physical, social, and environmental assets among others. The framework has broadened the scope of DRR by foregrounding health as a key concern, enhancing understanding of the interface between health and disaster risks. It also highlights concerns on human health and well-being that are common to DRR, climate change and sustainable development (12). There are several frameworks that already exist in growing awareness of the health implications of climate change as a health crisis. Initiatives such as Planetary Health, a solutions-oriented, multidisciplinary and social movement focused on addressing the impacts of destabilized natural systems on human health (13).

Healthcare systems are foundational for individual and community-level resilience. These systems play a critical role as the first line of defense in preventing potential adverse health outcomes only when they are: accessible, affordable, accountable, and equipped with reliable health services (14). Ensuring the preparedness of healthcare systems toward climate is essential for protecting at-risk populations through public health adaptation and resilience. Functional and competent healthcare systems depend on a range of stakeholders including governments, regulatory bodies, community health workers, and other healthcare. Healthcare is an indispensable component of broader climate action strategies which are positioned at the forefront of safeguarding and promoting public health (3). Beyond treatment and management, health systems connect with other key sectors such as disease prevention, public health risk management, emergency response, and healthcare service delivery to at-risk populations (15).

Understanding how health systems are adapting to climate challenges is pivotal for formulating evidence-based strategies that align with the core principles of health system resilience outlined in the new World Health Organization’s (WHO) operational framework (16). The new WHO framework is named ‘Operational Framework for Climate Resilient and Low Carbon Health Systems’. Its goal is to increase the climate resilience of health systems, protect and improve the health of communities in an unstable and changing climate, while optimizing the use of resources and implementing strategies to reduce greenhouse gasses (GHG) emissions (17). The framework emphasizes a dual responsibility, building health systems that can withstand climate-related shocks, while simultaneously reducing their carbon footprint (18). Mapping climate adaptation strategies is essential to foster evidence-based decision-making, identify best practices, and address gaps and challenges in health systems amidst the impact of climate change (19). Furthermore, climate change is one of the biggest threats to global health and primary health care (PHC). While in Africa, building and sustaining climate resilient PHC is a challenge as there is little evidence to inform health systems and policymakers (20).

This opinion examines the stresses, barriers, and challenges that healthcare systems in Africa face in adapting to the climate change crisis. Drawing on insights from the Climate Adaptation Research Program (CARP) projects, which brought together multidisciplinary researchers and disaster risk science experts from across the continent. It underscores the urgent need to address the impacts of climate change on healthcare systems. The authors aim to contribute to the development of robust, context-specific policies that strengthen the resilience of healthcare systems, particularly in African countries where adaptation efforts encounter significant hurdles. In this opinion paper, we discuss climate change impacts on disease burden, population displacement and public healthcare access, and public health facility vulnerability. Moreover, we also provide a case study from Uganda to illustrate healthcare vulnerability assessment. We end by discussing public health facility preparedness, public healthcare systems resilience and the concluding remarks.

### Climate change impacts on disease burden

According to the recent Intergovernmental Panel on Climate Change (IPCC), the leading international body for the assessment of climate change, climate risks are escalating faster with greater severity than anticipated due to global warming trends (21). The report reveals that 3.6 billion people are already living in highly vulnerable areas. Despite contributing minimally to global emissions, low-income countries and small island developing states (SIDS) are enduring the harshest health impacts (22). In these vulnerable regions, the death rate from extreme weather events over the last decade was 15 times higher than in less vulnerable areas (23).

Climate change directly affects environmental determinants of health, such as clean air, safe drinking water, and sanitation, which are closely linked to increases in communicable diseases and, non-communicable diseases (NCDs), and injuries (24). More than 80% of the global population is at risk of vector- borne disease (25). Mosquito-borne diseases (MBDs), such as malaria, dengue, chikungunya and zika, are the largest contributor disease burden, particularly in Africa (5). Some of the mechanisms through which climate change affects the transmission of MBDs include: changes in the development of vectors and pathogens; change of habitats; prolonged transmission seasons; changes in geographic spread; changes in vector abundance and behaviors of hosts and reduced abundance of mosquito predators (26). Zika, chikungunya, and dengue virus transmission peaks at 26–29°C while malaria caused by *P. falciparum* in Africa (about 99.7% cases of malaria) transmission peaks at 25°C (27). Extreme rainfall similarly creates high risk of exposure to MBDs due to its damaging effects on mosquito control measures. Heavy rains after a drought with low precipitation fills containers, which are the prime breeding sites. Additionally, floods displace human hosts, enhance new pathogen reservoirs, and re-establish vector breeding sites. Exacerbating the problems are the effects of prolonged droughts, crop failures, and extreme weather events which lead to increased food insecurity and malnutrition especially in children. In turn this weakens immune resilience hence a heightened vulnerability to illnesses (28).

The health-related impacts of climate change are multifaceted, including death and illness from increasingly frequent extreme weather events (e.g., heatwaves, storms and floods), disruptions to food systems, and increases in zoonoses and food-, water- and vector-borne diseases, Mental health challenges are also rising due to these crises (29). Furthermore, climate change undermines many social determinants of health, such as livelihoods, equity and access to health care and social support structures (30). Both direct and indirect impacts of climate change are strongly mediated by environmental, social and public health determinants (Supplementary Figure 1).

These climate-sensitive health risks disproportionately affect vulnerable and disadvantaged groups including individuals with preexisting health conditions (31). The health and disease burden arising from climate change extends beyond Africa’s border presenting a global challenge. It is imperative to reduce disparities and address the emerging threats to ensure a coordinated response to the pressing disease burden.

### Populations displacement and public healthcare access

Displacement has a devastating impact on individuals’ wellbeing resulting to numerous challenges including economic hardship, food insecurity, restricted access to healthcare, and instances of discrimination (32). The climate crisis not only directly affects individual and entire community health but also increases the number of forcibly displaced people due to home destruction, economic vulnerability and uncertainty. Literature has shown that displaced populations, especially those in resource limited settings and poor public health infrastructure experience significantly higher morbidity and mortality than their pre-disaster status (33) This displacement places additional strain on already fragile healthcare systems (3). Climate -induced displacement increases competition for scarce resources such as water, food and arable land, escalating tensions and fueling conflicts. These conflicts often emerge in regions with existing governance challenges, further burdening healthcare systems struggling to serve displaced populations.

The United Nations Refugee Agency estimates that 21.5 million people are displaced annually by weather-related disasters (34). The most affected populations are typically socially and economically vulnerable groups. Mass population displacement challenges conventional healthcare service delivery, especially for services that require the communities to access centralized hospitals. Many migrants face delays in receiving healthcare due to the stressed nature of the facilities (35). In addition to barriers like financial inaccessibility and communication challenges, African countries also face crises at the intersection of national borders further complicating healthcare access for displaced persons. Despite the existing literature, there is need for research to determine and enhance intersectional national boundary preparedness. Addressing the triple burden of health and wellbeing calls for a more inclusive and resilient healthcare for refugees.

### Public health facility vulnerability

The IPCC defines vulnerability to climate change as the degree to which a health system is susceptible to, or unable to cope with the adverse effects of climate variability and change (36). Climate change activates health hazards, creating a higher demand for care hence impacting healthcare’s coping capacity. Moreover, it also intensifies existing workforce shortages by increasing climate-driven illnesses and healthcare workers displacement, compromising delivery, especially in regions with weak healthcare systems in the continent (37). Due to the variations in how healthcare systems are organized and perform in Africa, it is also necessary to identify system-level interventions to enhance environmental sustainability of the healthcare system (38). Infrastructure systems, which are essential to the operation of healthcare facilities, do not function in isolation but are interdependent (39). During a disaster event, health care facilities are expected to operate efficiently to provide sufficient care for the sick or injured. However, this capacity is compromised when facilities lack reliable electricity, consistent water supplies and good road networks. These deficiencies significantly limit the ability of healthcare facilities to respond to emergencies and provide adequate care during crises.

#### Uganda-case study_ vulnerability assessment

Uganda is landlocked country in east Africa bordered by the Democratic Republic of the Congo (DRC), Kenya, Rwanda, South Sudan, and Tanzania.^1^ It is the Africa’s largest refugee-hosting country and ranks fifth globally in this regard (40). Uganda is highly vulnerable to climate change, facing increased droughts in the northern region, floods and unpredictable rainfall patterns. According to the Global Adaptation Index Rankings, Uganda is the 36th most vulnerable country to climate change and ranks 163rd in readiness to address its impacts.^2^

Uganda became the second country, after Nepal, to unveil a Climate Health Adaptation Plan, the vulnerability of its healthcare facilities to extreme weather events highlights significant risks to essential health infrastructure. A recent Health Sector Vulnerability and Adaptability Assessment (VAA) revealed that approximately 50% of healthcare facilities are vulnerable to drought, 40% to flooding, and nearly 32% to storms (Supplementary Figure 2). The impacts of events are extensive, affecting; water, sanitation and hygiene (WASH) systems, the stability of healthcare workforce, and the availability of medical technologies and supplies (41).

According to this assessment, climate change impacts all aspects of health and wellbeing in the country. Facilities in drought-prone areas often experience critical water shortages, compromising sanitation and hygiene practices which are essential for maintaining healthcare quality. The inadequacy of existing capacity and infrastructure underscores the urgent need to adopt strategies for building resilient health infrastructure. Ensuing healthcare remains operational and can effectively respond to climate-induced health risks is crucial for safeguarding public health.

### Public health facility preparedness

Health emergency and disaster preparedness is defined as the knowledge and capacities of health system to effectively anticipate, respond and recover from the impacts climate change (42). The African continent has experienced a significant increase in climate-related health emergencies over the last 20 years and these numbers are expected to rise further (43). Public health systems play a critical role in preparing communities to respond to and recover from climate related crises and emergencies. The public health consequences of disasters and emergencies initially affect local jurisdictions, highlighting the importance of localized public health preparedness. The Centers for Disease Control and Prevention (CDC) has established a vital framework for state, local, tribal, and territorial standards for public health preparedness planning (44). Additionally, the CDC supports regional centers to enhance the effectiveness and resilience of public health systems, promoting equitable access to services and resources. This approach aims to prevent disproportionate impacts on populations at higher public health risk (45).

On the African continent, fragile socioeconomic conditions and the lack of stable containment framework exacerbate the burden on public health system (46). Studies done in Kenya and Uganda revealed that many health facilities are unprepared to handle the growing burden of mosquito borne diseases (41, 47). Despite the increasing cases of dengue fever and malaria transmitted by *aedes aegypti and anopheles* spp. respectively, the existing health facilities are not capable of containing this rising crisis Building and improving health facility preparedness requires deliberate, multisectoral collaboration across African continent.

### Public healthcare systems resilience

Resilient health systems are those that can effectively prevent, prepare for, detect, adapt to, respond to, and recover from public health threats (14). Recognition of the Sendai Framework for Disaster Risk Reduction has significantly accelerated efforts to promote resilience in health systems and cross-field initiatives, such as Health Emergency and Disaster Risk Management (48). The COVID-19 pandemic highlighted the need for sustainable and resilient healthcare systems to protect population health. This requires consistently measuring the relative progress of health systems toward becoming more sustainable and resilient.

When health systems are resilient, they can effectively reduce the negative health and social impacts of climate change. However, as climate hazards become more frequent and severe, health systems face increasing threats. These challenges include heightened demand for services, strained infrastructure and supply chains, and pressure on the workforce (49).

Significant research has focused on the environmental sustainability of healthcare facilities, less emphasis has been placed on sustainably building climate resilience health systems, particularly in low- and middle-income countries (LMICs). The 2011 Public Health Preparedness Roadmap offers a structured approach for public health agencies to identify priorities and develop effective public health emergency preparedness and response programs. This roadmap serves as a critical tool for enhancing health system resilience in the face of escalating climate risks (Supplementary Figure 3).

To facilitate better understanding and monitoring of the Africa’s healthcare system’s relative weaknesses and strengths, and empower policy-makers to design interventions that improve its resilience and sustainability, a healthcare system sustainability and resilience index (HSSRI) can should be applied, HSSRI measures: (i) health system governance, (ii) health system financing, (iii) health system workforce, (iv) medicines and technologies, (v) health service delivery, (vi) population health and social determinants, and (vii) environmental sustainability (50). Sustainability measures such as system-level changes in healthcare supply and consumption should be adhered to.

## Conclusion

The impacts of climate change on health represent a significant global challenge, demanding the establishment of robust and resilient healthcare systems. To mitigate the catastrophic and lasting effects of the climate crisis and to prevent millions of climate-related deaths, it is essential to enhance the preparedness of healthcare systems. The limited integration of climate change considerations into other critical healthcare sectors restricts the ability of developing nations to adopt comprehensive adaptation and resilience strategies. By embedding adaptation strategies for resilience into healthcare planning and fostering cross-sectoral collaboration, Africa can better protect the health of its populations from climate impacts. There is an urgent need for applied research to evaluate climate change parameters and inform effective interventions. However, even in the absence of comprehensive evidence, immediate action is necessary. Waiting for complete scientific consensus risks exacerbating the impacts of climate change, potentially resulting in greater harm to lives and livelihoods in the future. Proactive measures must be prioritized to safeguard health and build climate-resilient systems. The ripple effect and burden of climate change on the healthcare systems calls for the activation and inclusion of multi-stakeholder platforms (MSPs) and cross border engagements which play a key role in DRR. This model improves the coordination between stakeholders working at different levels, supporting technical and financial capacities. Stakeholders’ engagement also allows for involvement of actors at different levels with different agendas, while creating space for participation and collaboration.

## Footnotes

^1^ https://youtu.be/CK3ymnCmqjM

^2^ https://gain.nd.edu/our-work/country-index/rankings/
