# Supplementary material for: The stifling burden of climate change on African public healthcare systems
Source: Front Public Health. 2025 May 30;13:1559737. doi: 10.3389/fpubh.2025.1559737 (PMC12162508; doi:10.3389/fpubh.2025.1559737)
Supplement: Supplementary file 1 [file Data_Sheet_1.docx]

Supplementary Material

**Supplementary Figure 1.**


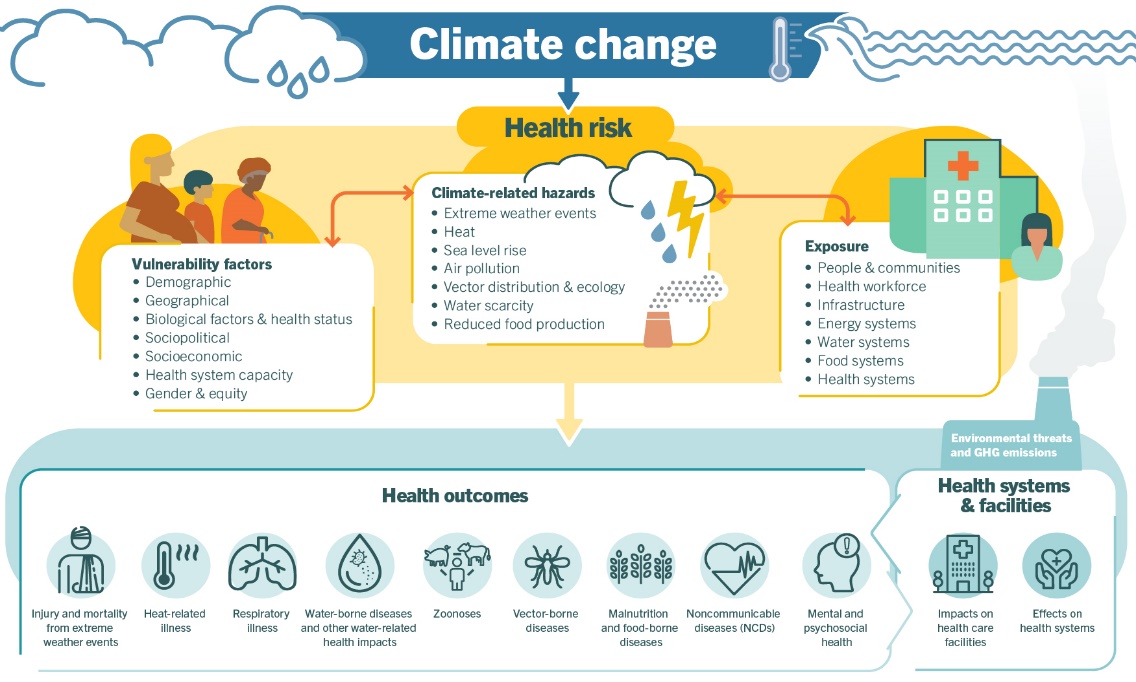


Figure 1: Climate-sensitive health risks, exposure pathways and vulnerability factors (WHO, Climate change and Health).


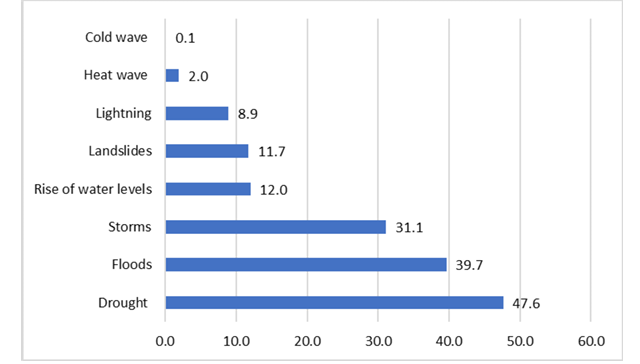
**Supplementary Figure 2.**

Figure 2: Climate Change Hazards or exposure to healthcare facilities (Uganda Climate change health vulnerability and adaptation assessment (VAA) report.

**Supplementary Figure 3.**


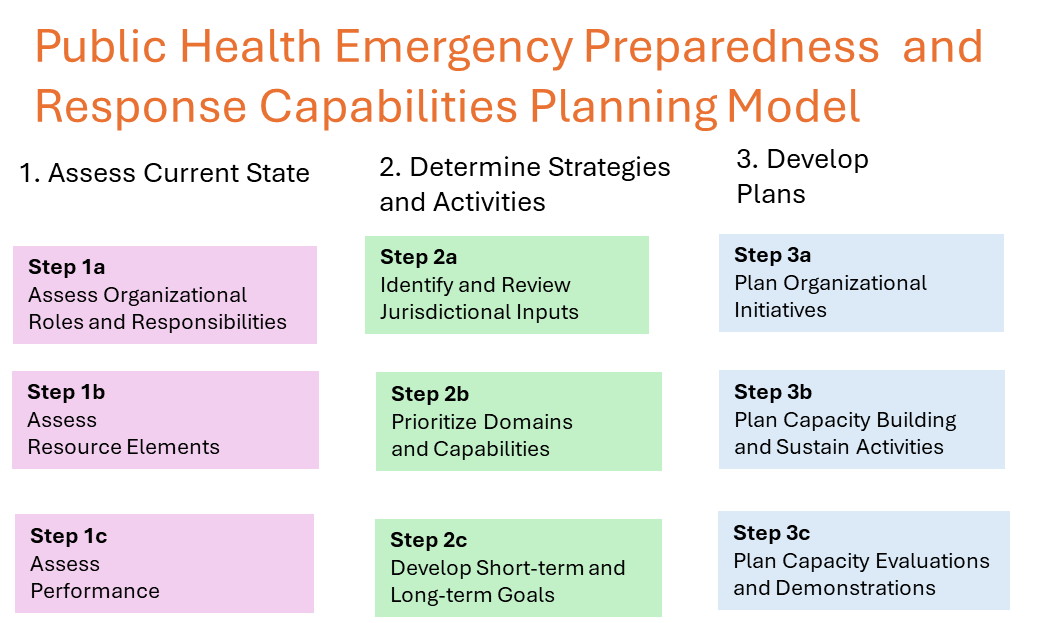


Figure 3: A three-phase approach to identify priorities emergency preparedness planning and response initiatives (CDC, Public Health Emergency Preparedness and Response).
